# Assessment of Pediatricians’ and General Practitioners’ Knowledge and Practice Regarding Oral Health, Dental Caries and Its Prevention in Children: A Cross-Sectional Study

**DOI:** 10.3390/dj11110259

**Published:** 2023-11-06

**Authors:** Antonija Tadin, Karmela Dzaja

**Affiliations:** 1 Department of Restorative Dental Medicine and Endodontics, Study of Dental Medicine, University of Split School of Medicine, 21000 Split, Croatia; 2 Department of Oral and Maxillofacial Surgery, Clinical Hospital Centre Split, 21000 Split, Croatia; 3 Study of Dental Medicine, University of Split School of Medicine, 21000 Split, Croatia

**Keywords:** dental caries, knowledge, oral health, physicians, pediatricians, prevention

## Abstract

Objectives: This study aimed to evaluate and compare the knowledge and practices of family physicians (general practitioners) and pediatricians concerning children’s oral health. Materials and Methods: This research involved a cross-sectional survey with 446 respondents, consisting of 77.8% women and 22.1% men, with 81.6% being general (family) practitioners and 18.4% pediatricians. The survey comprised five sections, gathering information on participants’ sociodemographic characteristics, routine oral cavity examination in clinical practice, knowledge about dental caries and its prevention, teething symptoms, and alternatives to fluorides for preventing dental caries. Results: The findings revealed an overall poor understanding of dental caries and its prevention, with an average score of 5.1 ± 1.6 out of a possible 10 points. Notably, practitioners with fewer pediatric patients during the workday, no training on oral health, and uncertainty about physicians’ active role in oral health prevention exhibited lower knowledge levels (*p* ˂ 0.05). Over 90% of participants conducted dental and oral mucosal examinations on their patients. About 25% had received continuing education on children’s oral health, and 70.6% expressed interest in further education on the subject. Conclusions: This study highlights insufficient knowledge among physicians regarding dental caries and its prevention. With most participants eager to learn and actively promote children’s oral health, providing training is essential to boost their knowledge and support children’s oral health.

## 1. Introduction

Oral health takes a critical role in the overall well-being of humans and is especially important during childhood. Children’s oral health affects their immediate well-being and has long-term consequences, including links to general health and effects on quality of life. The World Health Organization (WHO) recommends several ways to promote oral health as part of overall healthcare. By understanding the importance of oral health, addressing the challenges, and implementing effective strategies, we can encourage children to develop good oral habits that will last them a lifetime and ensure their overall well-being [1,2]. Primary care professionals, especially physicians, can significantly improve their patients’ oral health as part of their general healthcare [3,4,5,6,7,8].

Dental caries is one of the most common chronic diseases in childhood: about a quarter of preschool-aged children have caries in their primary teeth, while at least one in six children aged 6 to 11 years have caries in their permanent teeth. Tooth decay is a progressive disease that can be prevented if treated early. However, if left untreated, it worsens over time [2,3,4]. Neglected dental caries can significantly affect a child’s health and well-being, leading to problems such as pain, difficulty in eating and chewing, weight and growth problems, decreased self-confidence, and communication problems [1,3,4,5]. 

Oral hygiene, plaque control, oral health and nutrition education, fluoride or similar remineralizing agents, and regular dental check-ups are critical in caries prevention [9,10,11,12]. Parents should start oral hygiene before the eruption of the first deciduous tooth by using gauze soaked in saline solution. After teething, gradually introducing a toothbrush and fluoride toothpaste into the routine is recommended. Most guidelines advise parents to brush their children’s teeth and monitor brushing until children are ten years old [10]. The majority of dental associations suggest that a child’s first dental visit should occur within six months of the eruption of the first primary tooth and no later than 12 months of age. Some sources suggest that the optimal time for the first visit is between 12 and 18 months of age. Regular dental check-ups should take place every six months [11].

Internationally, there is an increasing emphasis on the role of non-dental personnel in improving oral health, particularly in children. The WHO recognizes the importance of oral health in interprofessional primary care practice [4,5,13,14]. Primary care physicians, including family physicians (general practitioners) and pediatricians, initiate contact with young children and their parents early on. They serve as a dependable source of information from birth, offering parents valuable and trustworthy guidance on preventing caries and other oral diseases in infants and young children. Enhanced participation of pediatricians and family physicians in oral health matters, particularly for those without access to dental care, plays a crucial role in ensuring that all patients receive pertinent oral health information and support [5,7,14,15,16,17]. Unfortunately, pediatricians and family physicians (general practitioners) are not adequately educated about oral health during their training and professional practice [14]. 

A worldwide study examined pediatricians’ knowledge, practice, and experience in oral care and prevention. The results revealed that in crucial areas, such as early clinical signs of dental caries, the recommended age for the first dental visit, the transmission of bacteria from mother to child as a cause of dental caries, and the suggested use of fluoride, pediatricians have limited knowledge [4,13,14,15,16,17,18]. There are also numerous barriers for pediatricians and family physicians to practice oral health. These include a lack of adequate education and training, time constraints on their practice, lack of clear referral pathways, and cost implications often complicated by health and dental insurance differences. Therefore, health authorities must recognize these gaps and implement appropriate action plans to improve education and training and to overcome other barriers. That is the only way to achieve a greater involvement of pediatricians and other primary healthcare professionals in children’s oral health [4,5,7,8,13].

This study aimed to evaluate and compare the knowledge and practices of family physicians (general practitioners), and pediatricians regarding oral health, dental caries, and its prevention in children. The specific objectives of the study were as follows: (1) to evaluate and compare family physicians (general practitioners) and pediatricians’ knowledge about the causes of dental caries, methods of oral hygiene, caries preventive measures and signs/symptoms of dental eruption; and (2) to evaluate and compare their practices in the treatment of oral diseases and dental caries in children. In this study, two hypotheses were formulated. The first hypothesis suggests that family physicians (general practitioners) and pediatricians have insufficient knowledge about the occurrence of dental caries and its preventive measures. The second hypothesis proposes that there is no significant difference between their practices in treating oral conditions and diseases in children and their knowledge about dental caries and its prevention.

## 2. Materials and Methods

This cross-sectional study was conducted from January to March 2023 at the Department of Restorative Dental Medicine and Endodontics, School of Medicine, Split. All respondents were duly informed about the purpose and objectives of the study. Completing and submitting the questionnaire was considered implicit consent to participate in this study. This study was ethically approved by the School of Medicine Ethics Committee and conducted following the latest guidelines and rules of the Declaration of Helsinki (2013). In addition, respondents’ rights and personal data were protected both during and after the study by the provisions of the Code of Medical Ethics and Deontology and the guidelines of STROBE (Strengthening the Reporting of Observational Studies in Epidemiology) [19].

### 2.1. Participants

This research used a questionnaire designed using the Google Forms^®^ application (Google, Mountain View, CA, USA). Respondents received the link to the questionnaire through the official email addresses on the websites of health centers in all counties of the Republic of Croatia. Participation in the study was voluntary and anonymous. The survey used a non-probabilistic sampling method involving family physicians (general practitioners) and pediatricians working in primary healthcare throughout Croatia. The questionnaire was sent to 1200 available email addresses. The respondents were encouraged to use the snowball method by forwarding the questionnaire to interested colleagues.

Inclusion criteria for participation included age of majority, and active employment in primary healthcare in the Republic of Croatia. The exclusion criteria included incomplete or inadequately completed questionnaires and failure to meet the inclusion criteria.

In 2021, the total number of healthcare teams in general practice/family medicine and preschool children by county in Croatia was 2616, of which 2333 were general practitioners, and 283 were pediatricians. In Croatia, pediatricians are responsible for the health of infants and preschool children, while general or family physicians take care of school children and the adult population [20]. The sample size required for the success of the study (n = 336) was calculated based on the total number of healthcare teams in the field of (general) medicine and preschool children in the Republic of Croatia (n = 2616). This calculation was performed using a 95% confidence interval and a 5% margin of error, utilizing the Sample Size Calculator by Raosoft, Inc., Seattle, WA, USA. 

### 2.2. Questionnaire

During the literature search, many studies were found, which were used to create the questionnaire [3,4,5,7,10,14,15,17,18,21,22,23]. Two specialists, one in endodontics with restorative dentistry and the other in pediatric and preventive dentistry, were involved in preparing the questionnaire. The questionnaire consisted of five parts and 40 close-ended questions (see Supplementary Materials). It took approximately 10 min to complete the questionnaire. All questions underwent a translation process from English to Croatian and then back-translation to English, ensuring the content validity and accuracy of the translation. Before the distribution of the questionnaire, a pilot study was conducted with 15 test group participants to ensure the transparency and appropriateness of the questionnaire, and the mentioned participants were not included in the final processing of the data. The internal consistency of the knowledge questions yielded a Cronbach’s coefficient alpha of 0.792.

The first part of the questionnaire was demographic and occupational. It consisted of eight questions about age, gender, educational level, specialty, workplace, years of experience, number of hours worked with patients, and number of pediatric patients per day. The second segment included 12 questions, all related to oral cavity examination in pediatric patients and their opinions regarding oral health practice in children. The questions related to the frequency with which respondents perform these examinations, the specialists to whom they would refer patients if they found mucosal changes, and why they refrain from performing these examinations in their daily clinical practice. Beyond the practical aspect, the participants also assessed their subjective understanding of oral health. They conveyed their view of their role as medical professionals in promoting oral well-being in children. The third part of the questionnaire included seven questions about the indicators and symptoms associated with the eruption of deciduous teeth. Symptoms experienced during tooth eruption included increased body temperature, diarrhea, nasal discharge, sleep disturbances, redness of the gums, excessive salivation, and decreased appetite. The respondents were offered “Yes”, “No”, “and “I do not know” response options for each of these manifestations. The fourth group comprised 10 questions and examined the participants’ dental health knowledge. The questions included etiological factors for the occurrence of caries, the influence of prolonged breastfeeding on the occurrence of caries, the impact of early childhood caries on the general health of children, and methods of oral hygiene, toothpaste, and the amount of fluoride used as a function of age. There was a correct answer to all ten questions. The sum of the correct answers was scored as the examinee’s total knowledge of the topic studied, with a maximum possible score of 10 points. According to Bloom’s threshold order, the participant’s overall knowledge was classified as good if the result was between 80% and 100% (8.0–10.0 points), moderate if the result was between 60% and 79% (6.0–7.9 points) and bad if the result was lower than 60% (<6.0 points) [24]. The fifth and final section consisted of only three questions aimed at evaluating the familiarity of the examined groups with alternative methods of caries prevention involving fluoride (understanding non-fluoride alternatives for dental caries prevention, the caries preventive effects of casein phosphopeptide, and the efficacy of pit and fissure sealants).

### 2.3. Data Analysis

The data were analyzed with the Statistical Package for the Social Sciences, version 26 (SPSS, IBM Corp, Armonk, NY, USA). The Kolmogorov–Smirnov test was used to assess the normality of the distribution of responses. Descriptive analysis was performed to determine the frequency and percentage of categorical data, whereas quantitative data were presented as means and standard deviations or medians and interquartile ranges. The chi-square test was used to compare the categorical variables between the two groups studied. In cases where one of the variables had a frequency of less than five, Fisher’s exact test was used. Multiple linear model (GLM) analysis was performed to identify the sociodemographic and oral health practice-related characteristics associated with the knowledge values. The statistical significance level was set at *p* < 0.05.

## 3. Results

A total of 446 physicians participated in the study, including 364 general practitioners (family physicians) and 82 pediatric specialists working in primary healthcare. The mean age of all participants was 43.9 ± 12.3 years (min = 26, max = 67 years; M_d_ = 44.0; IQR = 33.0–54.0). General practitioners (family physicians) averaged 42.0 ± 12.2 years of age (min = 26, max = 67 years, M_d_ = 42, IQR = 29.0–52.0), whereas pediatricians averaged 52.5 ± 8.5 years of age (min = 36, max = 64, M_d_ = 52.5, IQR = 44.0–62.0) (Table 1).

Table 2 shows the distribution of the responses to the questions about respondents’ practices and educational background related to children’s oral health. Among the participants, 82.1% of family physicians (general practitioners) and 94.0% of pediatricians perform tooth examinations on young patients. Regarding oral mucosa examinations, 97.1% of all respondents addressed the issue. Their importance as physicians in promoting oral health in children is recognized by 74.5% of general practitioners, while a slightly more significant proportion, 84.1% of pediatricians, hold the same view. A remarkable consensus can be seen in the recommendation of dental examinations, with almost all respondents, namely 98%, supporting this approach.

Table 3 shows the participants’ responses regarding how much they associate specific symptoms and signs with the eruption of deciduous teeth. A significant majority of the respondents related these signs to redness of the gums (91.5%), increased salivation (96.0%), and disturbed sleep patterns (95.5%).

Table 4 shows the results of questions assessing the respondents’ understanding of oral health and hygiene in children. Of note, only 37.9% of respondents knew that cariogenic bacteria could be transmitted from mother to child, and only 26.3% of respondents knew about the possible association between breastfeeding longer than one year and early childhood caries. In addition, only a small proportion of respondents correctly answered the question, “The recommended age at which children can cease parental-supervised toothbrushing” (4.2%).

The average score for the knowledge level among all respondents regarding children’s oral health was 5.1 ± 1.6 points out of a possible 10 (minimum 1, maximum 9; Md = 5, IQR = 4.00–6.00). Family (general) physicians exhibited an average knowledge score of 4.8 ± 1.5 points (min = 1, max = 9; Md = 5, IQR = 4.00–6.00), while pediatricians displayed a higher level of 6.1 ± 2.0 (min = 2, max = 9; Md = 6, IQR = 7.00–9.00). A statistically significant difference was observed between these two groups (*p* ≤ 0.001, Mann–Whitney U test). 

In contrast to general practitioners, pediatricians showed greater familiarity with fluoridation and alternative methods of remineralizing teeth. About 19.5% of the respondents (general practitioners/family physicians: 17.3%; pediatricians: 29.3%) knew about alternative forms of fluoridation in prevention, while 50% of physicians were aware of the effectiveness of pit and fissure sealants in preventing caries (general practitioners/family doctors: 46.7%; pediatricians: 64.6%). An even smaller proportion, only 9.9% of physicians, were familiar with the effect of casein phosphopeptides and amorphous calcium phosphate in caries prevention. This knowledge was distributed as 7.1% among general practitioners and 22.0% among pediatricians.

After adjusting for participant characteristics, a higher theoretical knowledge could not be associated with different sociodemographic characteristics. Only lower knowledge was associated with fewer pediatric patients per day (*p* = 0.010) (Table 5).

Lower knowledge can be correlated with the respondents’ average self-assessed knowledge (*p* = 0.004), lack of training on oral health in children (*p* = 0.045), and uncertainty about whether physicians should be actively involved in preventing oral health in children (*p* = 0.042) (Table 6).

## 4. Discussion

Family physicians, general practitioners, and pediatricians are likelier to see newborns and children than dentists, giving them valuable insight into overall health, including oral health. They are the first point of contact for educating parents about children’s oral health, preventing oral diseases, and guiding the treatment of oral diseases [5,23]. This study aimed to evaluate the knowledge and practices of family physicians (general practitioners) and pediatricians regarding dental caries and prevention methods. The research hypothesis assumed that the studied population does not have sufficient knowledge about the occurrence of dental caries and its preventive measures - this hypothesis was confirmed. The average knowledge of all respondents on the ten questions asked was poor, with an average score of 5.1 ± 1.6. General practitioners (family physicians) had a slightly lower knowledge level than pediatricians. A higher level of knowledge has been associated with seeing more pediatric patients per day, oral health education in children, and an awareness of their active role in preventing oral health problems in children. It is important to highlight that the pediatric specialty program includes one-week training in the pediatric dentistry department, a missing component in the family medicine specialty [25]. The respondents gave the best answers to the questions that caries is a chronic disease (79.6%), that advanced carious lesions cause irreversible damage to hard tooth tissue (83.0%), and that untreated caries in children can have an impact on overall health (89.0%). Respondents needed to be made aware of the effects of prolonged breastfeeding on the incidence of dental caries (27.6%) and that toothpaste containing fluoride can be used at 6 to 12 months of age (11.2%) in an amount equal to a rice grain. In a study from Saudi Arabia, pediatricians also had higher knowledge (5.05 vs. 4.28), better attitude (2.38 vs. 2.30), and better practice (4.0 vs. 3.58) compared with primary care physicians [26]. However, another study, also from Saudi Arabia, showed no significant differences in knowledge, attitude, and practice between pediatricians and family physicians [7].

Most family physicians (82.1%) and pediatricians (94.0%) perform oral cavity and teeth examinations in children as part of routine examinations. A survey conducted in Bosnia and Herzegovina found that 87% of participants believed that dental examinations should be included in pediatric screening; however, only 36% of respondents performed them regularly [27]. In Saudi Arabia, a significant proportion of pediatricians (97.1%) examined children, but a substantial number of general practitioners (82.9%) also performed these examinations, which is consistent with our study [26]. Similar results were observed in a comprehensive European survey, in which 97% of respondents agreed that such examinations were essential [28]. In contrast, the results from Turkey were mixed: 85.7% of family physicians did not examine children’s oral cavities, and pediatricians did so only when problems occurred (44.8%) or at the mother’s request (31.0%) [29]. Another study from Saudi Arabia indicated that 43.6% of participants reported conducting routine examinations for children, with pediatricians (64.8%) demonstrating significantly better practice compared to family physicians (35.2%) [7]. Several factors contribute to the reluctance of pediatricians and general practitioners to perform oral examinations or treat oral lesions. Key factors include a lack of knowledge due to inadequate training and time constraints [7,8,13,14]. Many respondents in this study chose the “other” option, possibly to hide their lack of expertise or to cope with limited time and a shortage of specialists.

During their training and clinical practice, a substantial proportion of family physicians (79.7%) did not attend oral health courses, whereas half of the pediatricians studied (41 of 82) did. If such educational opportunities were available, a substantial proportion of family physicians (67.8%) expressed interest, and even more pediatricians (83.0%) were willing to participate. By comparison, a study conducted among pediatricians in Syria found that 92% of respondents had not received training on children’s oral health during their residency training, and almost all pediatricians (∼99.0%) felt that such training was necessary [3]. In Spain, a study found that only a tiny proportion of pediatricians (9.2%) received training in pediatric dentistry during their specialty training, and almost all respondents (98.1–98.3%) acknowledged the need for oral health training during medical school and after graduation [30].

According to the different guidelines, the first dental visit should occur six months after the first eruption of a deciduous tooth, usually in the first year of a child’s life [11]. A small percentage of primary care physicians who agreed to participate (∼10%) reported advising parents of pediatric patients to have the first dental examination within the child’s first year of life. Less than half of pediatricians, 44.0%, recommended that children receive a dental examination during the first year of life. Slightly more than half of primary care physicians (∼54.0%) and exactly half of pediatricians suggest that parents schedule the first examination during the child’s third year of life, which is not consistent with the established guidelines. The respondents may not adequately emphasize the significance of dental visits to their young patients and parents, likely stemming from a lack of familiarity with guidelines and educational resources. Improving this situation requires a national-level promotion of oral health and enhanced education for pediatricians and general practitioners. A survey of pediatricians in Syria found that one-third (34.9%) recommend the first dental examination in the child’s first year of life. In contrast, a survey from Spain showed a slightly higher percentage, with 44% of pediatricians advising parents correctly [3,30]. A study conducted in Saudi Arabia demonstrated that 47.6% of family physicians and 42.2% of pediatricians recommend parents have their first visit to the dentist within the child’s initial 12 months of life [26].

Less than half of all respondents (44%), namely one-third of family physicians (37.9%) and twice as many pediatricians (73.1%), were aware of the possibility of transmission of cariogenic bacteria from mother to child. Comparing these results with those of other studies, a study from Saudi Arabia found that 45% of family physicians and slightly more than half of pediatricians (54.8%) had this knowledge [26]. A survey conducted in Turkey, which included family physicians and pediatricians, showed that nearly one-third of family physicians (32%) and a much higher proportion of pediatricians (88%) were aware of this mode of transmission [29]. The research results from Qatar are devastating, with 84% of respondents who participated in the study conducted among general practitioners and pediatricians being unaware of the possibility of bacterial transmission [18].

Numerous studies have found an association between prolonged breastfeeding beyond 12 months of age and an increased incidence of dental caries and poorer overall oral health. This association may be attributed to a combination of factors, including prolonged breastfeeding, particularly at night, food introduction, and poor oral hygiene compliance [30,31,32]. Many respondents would like more education about the link between prolonged breastfeeding and dental caries. More than half of family physicians (51.6%) and of pediatricians (60.9%) believe that the age at which a child stops breastfeeding is not a significant factor leading to tooth decay. Similar results were obtained from studies in Syria, where only 15% of respondents believe that prolonged breastfeeding can lead to caries, and a remarkable 67.7% of pediatricians in Spain do not consider breastfeeding beyond the indicated duration to be an etiological factor for caries [3,30].

The tooth brushing technique for children is influenced by coordinated muscle movements and the developmental level of motor skills. Parental supervision of children’s tooth brushing consists of observing and guiding them in the proper tooth brushing technique. It is recommended that parents supervise tooth brushing for children between the ages of seven and nine, using the National Health Service guidelines as a guide. This age range is considered appropriate because children of this age usually have sufficient motor skills for independent toothbrushing [3,33,34]. Only a small fraction of respondents (4.2%) knew the correct answer regarding the age until which children should be supervised. The results from a study in Syria revealed that more than half of the respondents (57.6%) believed parents should supervise brushing until the child becomes independent, regardless of chronological age. Additionally, the same study indicated that 37.5% believed children should be monitored up to 7–8 years of age. In Spain, 60% of pediatricians considered this age range appropriate for supervision [3,30].

According to the National Health Service recommendation, children between the ages of three and six should brush their teeth with a pea-sized amount of toothpaste containing 1000 ppm fluoride. Family physicians included in the study answered the above statement correctly 44.5% of the time, while pediatricians answered correctly 62.2% of the time. A Turkish study involving family physicians and pediatricians found that 90% of respondents educated parents about the importance of fluoride toothpaste, and among pediatricians, this figure was as high as 100% [29]. In Syria, more than three-quarters of pediatricians (81%) did not know the recommended amount of fluoride toothpaste for children under six years of age, while only a small number (4.8%) did [3]. Of the pediatricians examined in a study conducted in Spain, 52.9 % knew the correct answer [30].

Silver Diamine Fluoride (SDF) is a minimally invasive topical solution used in early interventions and tooth decay prevention. Applied directly to affected teeth, it halts cavity progression and strengthens the enamel. SDF shows promise in managing dental caries in children, especially when traditional restorative methods may be challenging. While traditionally used by dentists, its straightforward application without the need to remove caries makes it a potential option for physicians and pediatricians. However, regulatory permissions may be required, subject to individual country regulations [35].

The eruption of deciduous teeth is a significant milestone in the life of infants, accompanied by various signs and symptoms that can be both local and systemic. Local symptoms include red and tender gums, increased salivation, and eruption cysts. Systemic symptoms include irritability, fever, loss of appetite, restless sleep, tearfulness, and even diarrhea. It is worth noting that these symptoms may vary from baby to baby, and it is still unclear whether the eruption of deciduous teeth directly causes the disorders or whether the disorders coincide with the eruption process [36,37]. More than 90% of respondents associated teething with increased salivation, rubbing of the gums, and sleep disturbances. Similar results were also obtained from studies conducted among physicians and pediatricians in the USA, Iran, and Turkey [36,38,39].

This study found that general practitioners and pediatricians performed regular oral cavity examinations in children in more than 80% of the cases. A significant majority of the study’s participants recognized the important role they play as healthcare providers in promoting children’s overall health (76%) and were committed to actively contributing to improving children’s oral and dental health. Similar findings were obtained in studies from Egypt, where 94.3% of respondents recognized the critical role of pediatricians in promoting children’s oral and dental health, as well as from Ontario (87.3% of respondents) and Bosnia and Herzegovina (88.7% of participants) [27,40,41].

Molar-Incisor Hypomineralization (MIH) is a dental condition characterized by insufficient enamel quality and quantity in molars and incisors [42]. Its prevalence is increasing, ranging from 2% to 40% among children globally, posing a substantial oral health concern [43]. MIH’s multifactorial etiology includes genetic and environmental factors, such as maternal illness, cesarean delivery, delivery complications, premature birth, low birth weight, respiratory diseases, high fever, and early medication use. In addition to aesthetic problems, MIH is associated with an increased risk of dental caries and compromised oral health [44]. Despite its escalating prevalence and importance, no research on MIH knowledge among pediatricians and physicians exists. There is a pressing need for more studies and educational initiatives to raise awareness and knowledge among medical professionals. Recognizing the significance of MIH empowers pediatricians to contribute to its early diagnosis and management, ensuring improved oral health and overall well-being for the children under their care [45].

However, it is important to acknowledge certain limitations in this study. The sample size, the method of sampling respondents and the cross-sectional study design may not accurately represent all pediatricians and family physicians in Croatia. The closed questionnaire used in the study might not fully capture the nuanced aspects of participants’ knowledge, attitudes, and practices. For future research, it is recommended to conduct a qualitative study aimed at exploring the barriers, experiences, and underlying reasons for the suboptimal implementation of practices for preventing oral diseases and dental caries in children. This approach will offer a deeper understanding of the challenges and motivations involved, facilitating more targeted and effective interventions in the field of pediatric oral health. Additionally, including only those physicians with available email addresses on health center websites might also introduce a selection bias.

Indeed, as is evident from the information provided, family physicians and pediatricians play a crucial role in safeguarding the oral health of children. It is imperative to emphasize the need for comprehensive education, both in theory and clinical practice, focused on maintaining and preventing children’s oral health issues. This education should be an integral part of the curriculum in medical schools and during specialized training, ensuring that healthcare professionals are well-equipped to address children’s oral health needs. Ultimately, prioritizing children’s oral health, incorporating appropriate education, and fostering collaboration between different medical disciplines are essential steps towards ensuring the well-being and future health of the younger generation.

## 5. Conclusions

This study revealed insufficient knowledge among general physicians and pediatricians concerning dental caries and its prevention. A firmer grasp of this subject was correlated with a higher number of child patients seen per day, education in the realm of oral health, and an awareness of their crucial role in preventing oral health issues in children. It is essential to highlight that most respondents were willing to receive education on this topic. Consequently, various dental and medical societies should take heed of this feedback and expand the range of educational opportunities on this subject.

## Figures and Tables

**Table 1:** Demographic and professional characteristics of the respondents.

Characteristics		Total	General/Family Physicians	Pediatricians	*p* Values
Gender	Female	347 (77.8)	270 (74.2)	77 (94.0)	≤0.001 *
	Male	99 (22.2)	94 (25.8)	5 (6.0)	
Age group (years)	<35	117 (26.2)	117 (32.0)	0 (0.0)	≤0.001 *
	35–44	120 (26.9)	98 (27.0)	22 (26.8)	
	45–54	104 (23.3)	77 (21.2)	27 (32.2)	
	>55	105 (23.5)	72 (19.8)	33 (40.0)	
Academic qualification	DM	416 (93.3)	338 (92.8)	78 (95.1)	0.313
	MSc	20 (4.5)	16 (4.4)	4 (4.9)	
	PhD	10 (2.2)	10 (2.8)	0 (0.0)	
Type of practice	Community healthcare practice	347 (77.8)	290 (79.7)	57 (69.5)	0.046 *
	Concession healthcare practice	99 (22.2)	74 (20.3)	25 (30.5)	
Working experience (years)	1–5	110 (24.7)	96 (26.3)	14 (17.1)	0.036 *
	6–10	84 (18.8)	73 (20.1)	11 (13.4)	
	11–20	119 (26.7)	96 (26.3)	23 (28.1)	
	>20	113 (29.8)	99 (27.2)	34 (41.4)	
Patient care per day (hours)	1–8	343 (79.6)	284 (78.1)	59 (72.0)	0.239
	>8	103 (23.1)	80 (21.9)	23 (28.0)	
Number of children patients per day	<10	263 (59.0)	263 (72.2)	0 (0.0)	≤0.001 *
	10–20	56 (12.6)	56 (15.3)	0 (0.0)	
	21–50	40 (9.0)	14 (3.8)	26 (31.7)	
	>50	87 (19.5)	31 (8.7)	56 (68.3)	

Data are presented as whole number and percentage. χ^2^—chi-square test or Fisher’s exact test, * *p* < 0.05.

**Table 2:** Oral-health-related practice and attitudes among respondents.

Oral-Health-Related Practice		Total	General/Family Physicians	Pediatricians	*p* Values
Conducting tooth examination	Yes	376 (94.3)	299 (82.1)	77 (94.0)	0.008 *
	No	70 (15.7)	65 (17.9)	5 (6.0)	
Conducting oral mucosa examination	Yes	433 (97.1)	351 (96.4)	82 (100)	0.082
	No	13 (2.9)	13 (3.6)	0 (0.0)	
Reason for not conducting oral examinations	Lack of time	57 (12.8)	49 (13.5)	8 (9.7)	0.340
	Lack of knowledge	21 (4.7)	19 (5.2)	2 (2.4)	
	Other	368 (82.5)	296 (81.3)	72 (87.9)	
Patients’ management with oral mucosal lesions	Yes	391 (87.7)	311 (85.4)	80 (97.6)	0.003 *
	No	55 (12.3)	53 (14.6)	2 (2.4)	
Reason for non-treatment of patients with oral mucosal lesions	Lack of time	18 (4.1)	14 (3.8)	4 (4.8)	0.073
	Lack of knowledge	42 (9.4)	40 (11.0)	2 (2.4)	
	Other	386 (86.5)	310 (82.5)	76 (92.8)	
Referral of patients with oral mucosal lesions	Dentists	205 (46.0)	181 (49.7)	24 (29.3)	≤0.001 *
	Oral medicine specialist	86 (19.3)	83 (22.8)	3 (3.7)	
	Pediatric dentist	7 (1.6)	2 (0.5)	5 (6.0)	
	Other	148 (32.2)	98 (27.0)	50 (60.1)	
Self-assessment of personal knowledge about oral health	Good/Excellent	186 (41.7)	147 (40.3)	39 (47.6)	≤0.001 *
	Poor/Very poor	158 (35.4)	144 (39.6)	14 (17.1)	
	Average	102 (22.9)	73(20.1)	29 (35.3)	
Perceived education on oral health topics during medical graduate and postgraduate studies	Yes	115 (25.8)	74 (20.3)	41 (50.0)	≤0.001 *
	No	331 (74.2)	290 (79.7)	41 (50.0)	
Interested in further education on the topic of oral health in children	Yes	315 (70.6)	247 (67.8)	68 (83.0)	0.011 *
	No	19 (4.3)	19 (5.3)	0 (0.0)	
	Do not know	112 (25.1)	98 (26.9)	14 (17.0)	
Physicians should actively participate in the prevention of oral and dental changes in children	Yes	340 (76.2)	271 (74.5)	69 (84.1)	0.096
	No	42 (9.4)	39 (10.7)	3 (3.6)	
	Do not know	64 (14.3)	54 (14.8)	10 (12.3)	
Referral patients to the dentist	Yes	437 (98.0)	355 (97.5)	82 (100.0)	0.150
	No	9 (2.0)	9 (2.5)	0 (0.0)	
Referral patients to the dentist—children’s age	<1 year	76 (17)	40 (10.9)	36 (44.0)	≤0.001 *
	>3 years	103 (23.1)	98 (26.9)	5 (6.0)	
	6 years	27 (6.1)	27 (7.4)	0 (0.0)	
	1–3 years	240 (53.8)	199 (54.8)	41 (50.0)	

Data are presented as whole number and percentage. χ^2^—chi-square test or Fisher’s exact test, * *p* < 0.05.

**Table 3:** Signs and symptoms of teething.

Signs and Symptoms		Total	General/Family Physicians	Pediatricians	*p* Values
Fever	Yes	355 (79.6)	304 (83.5)	51 (62.1)	≤0.001 *
	No	69 (15.5)	46 (12.6)	23 (28.1)	
	Do not know	22 (4.9)	14 (3.9)	8 (9.8)	
Diarrhea	Yes	222 (49.8)	176 (48.3)	46 (56.1)	0.321
	No	161 (36.1)	133 (36.5)	28 (34.1)	
	Do not know	63 (14.1)	55 (15.2)	8 (9.8)	
Runny nose	Yes	115 (25.8)	91 (25.0)	24 (29.2)	0.321
	No	265 (59.4)	215 (59.1)	50 (61.0)	
	Do not know	65 (14.8)	58 (15.9)	8 (9.8)	
Disturbed sleep	Yes	426 (95.5)	347 (95.6)	79 (96.3)	0.368
	No	10 (2.5)	7 (1.9)	0 (0.0)	
	Do not know	9 (2.0)	9 (2.5)	3 (3.7)	
Gum rubbing	Yes	408 (91.5)	336 (92.3)	72 (87.8)	0.351
	No	24 (5.4)	17 (4.6)	7 (8.5)	
	Do not know	14 (3.1)	11 (3.1)	3 (3.7)	
Increased salivation and drooling	Yes	428 (96.0)	349 (95.8)	79 (96.3)	0.035 *
	No	6 (1.3)	3 (0.82)	3 (3.7)	
	Do not know	12 (2.7)	12 (3.4)	0 (0.0)	
Loss of appetite	Yes	359 (80.5)	295 (81.1)	65 (79.2)	0.101
	No	49 (11.0)	35(9.6)	14 (17.1)	
	Do not know	37 (8.5)	34 (9.3)	3 (3.7)	

Data are presented as whole number and percentage. χ^2^—chi-square test or Fisher’s exact test, * *p* < 0.05.

**Table 4:** The frequency distribution (%) of physicians’ answers to the questions regarding children’s oral health—knowledge test.

Question		Total	General/Family Physicians	Pediatricians
Bacteria that cause decay can spread from mother to child [4,10]	*Yes*	198 (44.4)	138 (37.9)	60 (73.1)
	No	112 (25.1)	103 (28.2)	9 (11.0)
	Do not know	136 (30.5)	123 (33.9)	13 (15.9)
Advanced dental caries is a chronic condition [2,3,4,10]	*Yes*	355 (79.6)	283 (77.7)	72 (87.8)
	No	72 (16.1)	63 (17.3)	9 (11.0)
	Do not know	19 (4.3)	18 (5.0)	1 (1.2)
The development of carious lesions causes the destruction of dental hard tissue [2,3,4,17]	*Yes*	370 (83.0)	297 (81.5)	73 (89.1)
	No	21 (4.7)	17 (4.6)	2 (2.4)
	Do not know	55 (12.3)	50 (13.9)	7 (8.5)
Prolonged breastfeeding increases the risk of having dental caries [10]	*Yes*	124 (27.6)	96 (26.3)	28 (34.1)
	No	238 (53.4)	188 (51.6)	50 (60.9)
	Do not know	84 (18.0)	80 (22.1)	4 (5.0)
Untreated dental decay could affect the general health of the child [2,4]	*Yes*	397 (89.0)	318 (87.3)	79 (96.4)
	No	11 (2.5)	9 (2.4)	2 (2.4)
	Do not know	38 (8.5)	37 (10.3)	1 (1.2)
Initiating brushing for children’s teeth [10]	Eruption of all teeth	16 (3.6)	15 (4.1)	1 (1.2)
	Eruption of multiple teeth	102 (22.8)	100 (27.4)	2 (2.4)
	*First tooth eruption*	308 (69.1)	230 (63.2)	78 (95.2)
	Do not know	20 (4.5)	19 (5.3)	1 (1.2)
The recommended age at which children can cease parental-supervised toothbrushing [10]	3 years	198 (44.4)	170 (46.7)	28 (34.2)
	6 years	221 (49.6)	173 (47.5)	48 (58.5)
	*Prepuberty*	19 (4.2)	14 (3.8)	5 (6.1)
	Do not know	8 (1.8)	7 (2.0)	1 (1.2)
The recommended age for children to start using a rice grain-sized smear of fluoridated toothpaste [4,10,17]	*6–12 month*	50 (11.2)	37 (10.2)	13 (15.9)
	>3 years	202 (45.3)	155 (42.6)	47 (57.3)
	>6 years	98 (22.0)	86 (23.6)	12 (14.6)
	Do not know	96 (21.5)	86 (23.6)	10 (12.2)
The recommended amount of fluoride toothpaste for children aged 3 to 6 years [4,10,17]	Regular (1–2 cm)	13 (2.9)	6 (1.6)	7 (8.5)
	*Pea size*	213 (47.8)	162 (44.5)	51 (62.2)
	Grain of rice	115 (25.8)	100 (27.4)	15 (18.3)
	Do not know	105 (23.5)	96 (26.3)	9 (11.0)
The potential for remineralization of early stage carious lesions [10,17]	*Yes*	236 (52.9)	194 (53.2)	42 (51.2)
	No	75 (16.8)	54 (14.8)	21 (25.6)
	Do not know	135 (30.3)	116 (32.0)	19 (23.2)

Data are presented as numbers and percentages. Correct answers are italicized.

**Table 5:** Generalized linear model (GLM) analysis of the relationship between physicians’ sociodemographic characteristics in relation to knowledge regarding children’s oral health.

Characteristics		β	S.E.	95% Wald Confidence Interval		*p* Values
				Lower	Upper	
Gender	Male	−0.394	0.244	−0.873	0.084	0.106
	Female	References				
Age group (years)	<35	−0.540	0.487	−1.496	0.415	0.268
	35–44	−0.185	0.455	−1.078	0.708	0.685
	45–54	−0.670	0.392	−1.438	0.098	0.087
	>55	References				
Academic qualification	DM	1.347	0.722	−0.068	2.762	0.062
	MSc	0.812	0.858	−0.870	2.495	0.344
	PhD	References				
Type of practice	Community healthcare	−0.139	0.300	−0.728	0.449	0.643
	Concession healthcare	References				
Specialization	General/family physicians	−0.843	0.462	−1.749	0.063	0.068
	Pediatricians	References				
Working experience (years)	1–5	0.424	0.481	−0.520	1.369	0.378
	6–10	0.017	0.432	−0.830	0.864	0.968
	11–20	0.116	0.386	−0.641	0.873	0.764
	>20	References				
Patient care per day (hours)	1–8	−0.038	0.254	−0.537	0.461	0.880
	>8	References				
Number of children patients per day	<10	−0.316	0.415	−1.131	0.499	0.447
	10–20	−1.197	0.467	−2.114	−0.281	0.010 *
	21–50	0.157	0.515	−0.853	1.168	0.760
	>50	References				

Reference knowledge or confidence level category is “low”, * *p* < 0.05.

**Table 6:** Generalized linear model (GLM) analysis of the relationship between physician’s oral-health-related practice in relation to knowledge regarding children’s oral health.

Oral-Health-Related Practice		β	S.E.	95% Wald Confidence Interval		*p* Values
				Lower	Upper	
Conducting tooth examination	No	−0.158	0.293	−0.733	0.418	0.591
	Yes	References				
Conducting oral mucosa examination	No	0.971	0.672	−0.346	2.288	0.149
	Yes	References				
Patients’ management with oral mucosal lesions	No	0.097	0.349	−0.588	0.783	0.781
	Yes	References				
Self-assessment of personal knowledge about oral health	Poor/Very poor	0.618	0.324	−0.017	1.254	0.056
	Average	0.859	0.297	0.276	1.443	0.004 *
	Good/Excellent	References				
Perceived education on oral health topics during medical graduate and postgraduate studies	No	0.543	0.270	0.012	1.074	0.045 *
	Yes	References				
Interested in further education on the topic of oral health in children	No	−0.068	0.543	−1.133	0.998	0.901
	Do not know	0.062	0.245	−0.420	0.544	0.800
	Yes	References				
Physicians should actively participate in the prevention of oral and dental changes in children	No	0.336	0.373	−0.397	1.068	0.369
	Do not know	0.596	0.293	0.022	1.170	0.042 *
	Yes	References				
Referral patients to the dentist	No	0.603	0.692	−0.755	1.961	0.384
	Yes	References				

Reference knowledge or confidence level category is “low”, * *p* < 0.05.

## Data Availability

Data are available upon request from the corresponding author.

## Supplementary Materials

The following are available online at https://www.mdpi.com/article/10.3390/dj11110259/s1, File S1, Assessment of Pediatricians’ and General Practitioners’ Knowledge and Practice Regarding Oral Health, Dental Caries and its Prevention in Children—questionnaire.
