# Supplementary material for: Assessment of Pediatricians’ and General Practitioners’ Knowledge and Practice Regarding Oral Health, Dental Caries and Its Prevention in Children: A Cross-Sectional Study
Source: Dent J (Basel). 2023 Nov 6;11(11):259. doi: 10.3390/dj11110259 (PMC10670318; doi:10.3390/dj11110259)
Supplement: Supplementary file 1 [file dentistry-11-00259-s001.zip › dentistry-2602042-supplementary.pdf]

“Assessment of Pediatricians' and General Practitioners' Knowledge and Practice Regarding Oral Health, Dental Caries and its Prevention in Children – questionnaire”

I. Demographic and professional characteristics

1. Gender: □ Male □ Female
2. Age group (years): □<35 □ 35 – 44 □ 44 – 54 □ >55
3. Academic qualification: □ DM □ Master of Science □ Doctor of Philosophy
4. Specializations in medicine: □ General/family physicians □ Pediatricians
5. Type of practice setting: □ Community health care practice
   □ Concession health care practice
6. Working experience (years): □ 1-5 □ 6-10
   □ 11-20 □ ≤ 21
7. Number of children patients per day: □ <10 □ 11-20 □ 21-50 □ ≥51
8. Patient care per day (hours): □ 1-8 □ >8

II. Oral health-related practice and attitudes. Please mark the answer that best reflects your practice or opinion regarding the promotion of oral health in children.

9. Conducting tooth examination: □ Yes □ No
10. Conducting oral mucosa examination: □ Yes □ No
11. Reason for not conducting oral examinations:
    □ Lack of time □ Lack of knowledge □ Other
12. Patients’ management with oral mucosal lesions: □ Yes □ No
13. Reason for non-treatment of patients with oral mucosal lesions:
    □ Lack of time □ Lack of knowledge □ Other
14. Referral of patients with oral mucosal lesions: □ Dentists □ Oral medicine specialist
    □ Pediatric dentist □ Other
15. Self-assessment of personal knowledge about oral health: □ Excellent □ Good
    □ Average □ Poor
    □ Very poor
16. Perceived education on oral health topics during medal graduate and postgraduate:
    □ Yes □ No
17. Interested in further education on the topic of oral health in children:
    □ Yes □ No □ Do not know
18. Physicians should actively participate in the prevention of oral and dental changes in children:
    □ Yes □ No □ Do not know
19. Referral child patients to the dentist: □ Yes □ No
20. The age at which you typically refer a child patient for a dental examination:
    □ <1 year □ 1-3 years
    □ >3 years □ 6 years

III. Knowledge of signs and symptoms of teething. Please mark the answer that best reflects your opinion.

21. Fever: □ Yes □ No □ Do not know
22. Diarrhea: □ Yes □ No □ Do not know
23. Runny nose: □ Yes □ No □ Do not know
24. Disturbed sleep: □ Yes □ No □ Do not know
25. Gum rubbing: □ Yes □ No □ Do not know
26. Increased salivation and drooling: □ Yes □ No □ Do not know
27. Loss of appetite: □ Yes □ No □ Do not know

IV. Knowledge of oral health, dental caries and its prevention in children. Please mark the answer that best reflects your opinion.

28. Bacteria that cause decay can spread from mother to child:
□ Yes □ No □ Do not know
29. Advanced dental caries is a chronic condition:
□ Yes □ No □ Do not know
30. The development of carious lesions causes the destruction of dental hard tissue:
□ Yes □ No □ Do not know
31. Prolonged breastfeeding increases the risk of having dental caries:
□ Yes □ No □ Do not know
32. Untreated dental decay could affect the general health of the child:
□ Yes □ No □ Do not know
33. The potential for remineralization of early-stage carious lesions:
□ Yes □ No □ Do not know
34. The appropriate time to initiate tooth brushing for children:
□ Eruption of all teeth
□ Eruption of multiple teeth
□ First tooth eruption
□ Do not know
35. The recommended age at which children can cease parental-supervised toothbrushing:
□ 3 years □ 6 years
□ Prepuberty □ Do not know
36. The recommended age for children to start using a rice grain-sized smear of fluoridated toothpaste: □ 6-12 month □ >3 years
□ >6 years □ Do not know
37. The recommended amount of fluoride toothpaste for children aged 3 to 6 years:
□ Regular (1-2 cm) □ Pea size
□ Grain of rice □ Do not know

V. An alternative to fluorides in caries prevention. Please mark the answer that best reflects your opinion.

38. Knowledge of non-fluoride alternatives for dental caries prevention: □ Yes □ No
39. Knowledge of the caries preventive effects of casein phosphopeptide: □ Yes □ No
40. Knowledge of the efficacy of pit and fissure sealants as caries preventive measures:
□ Yes □ No
